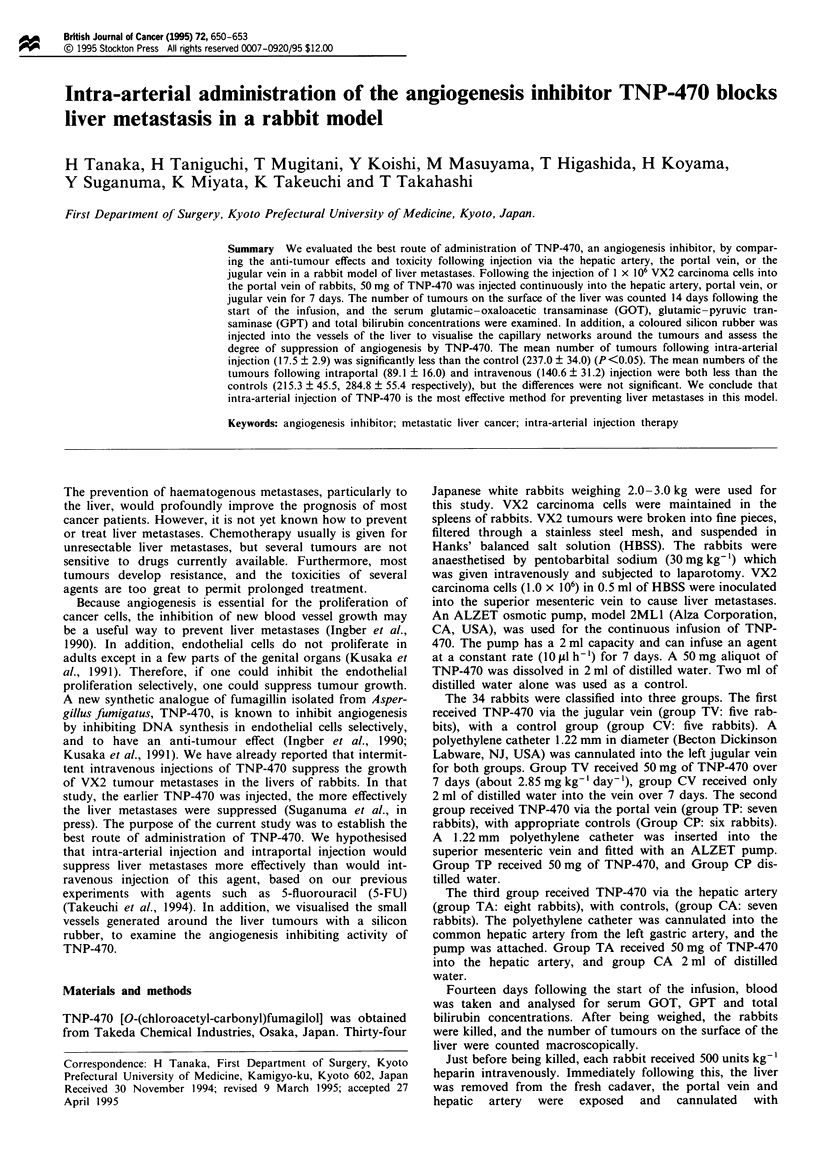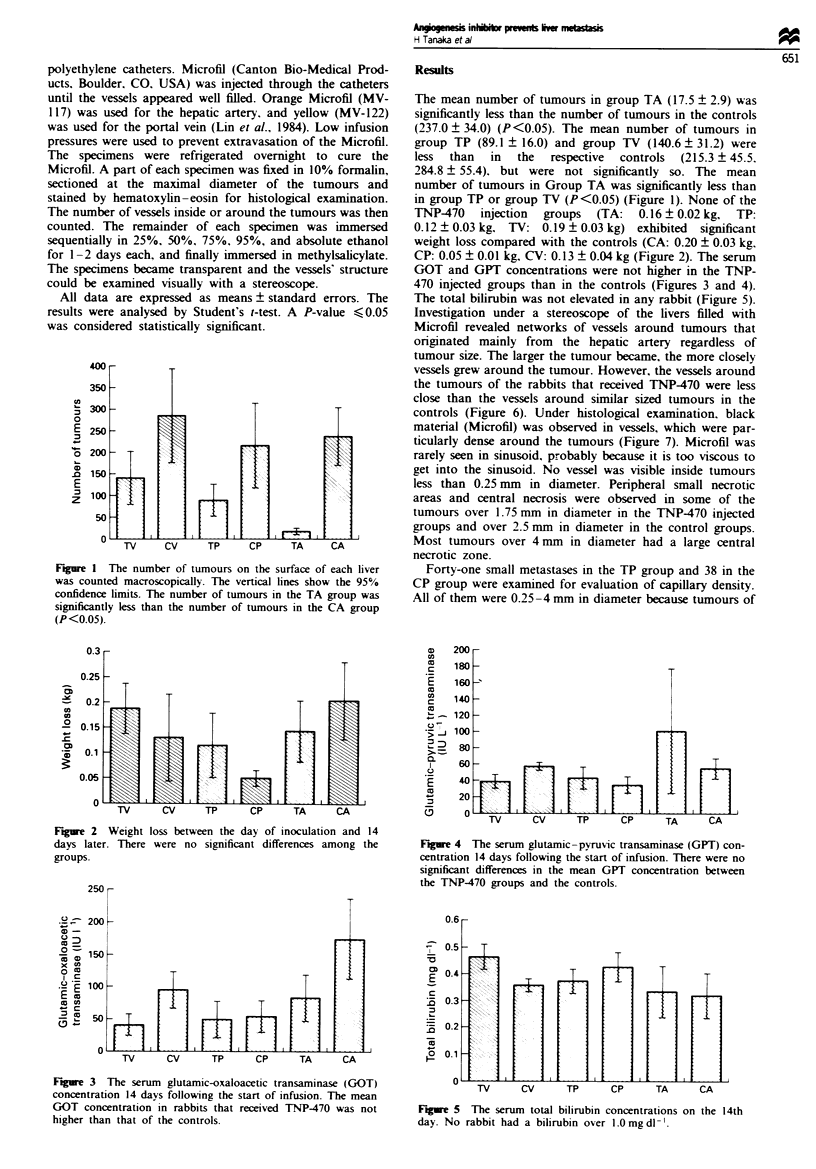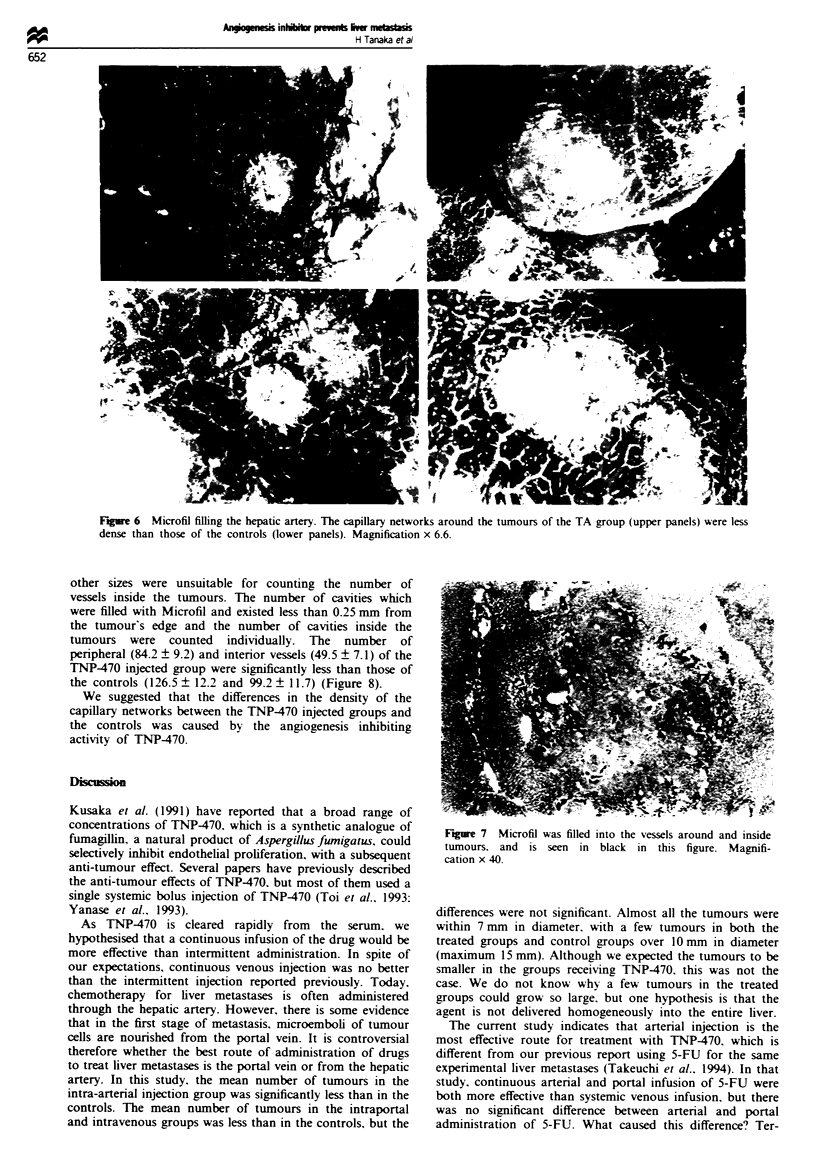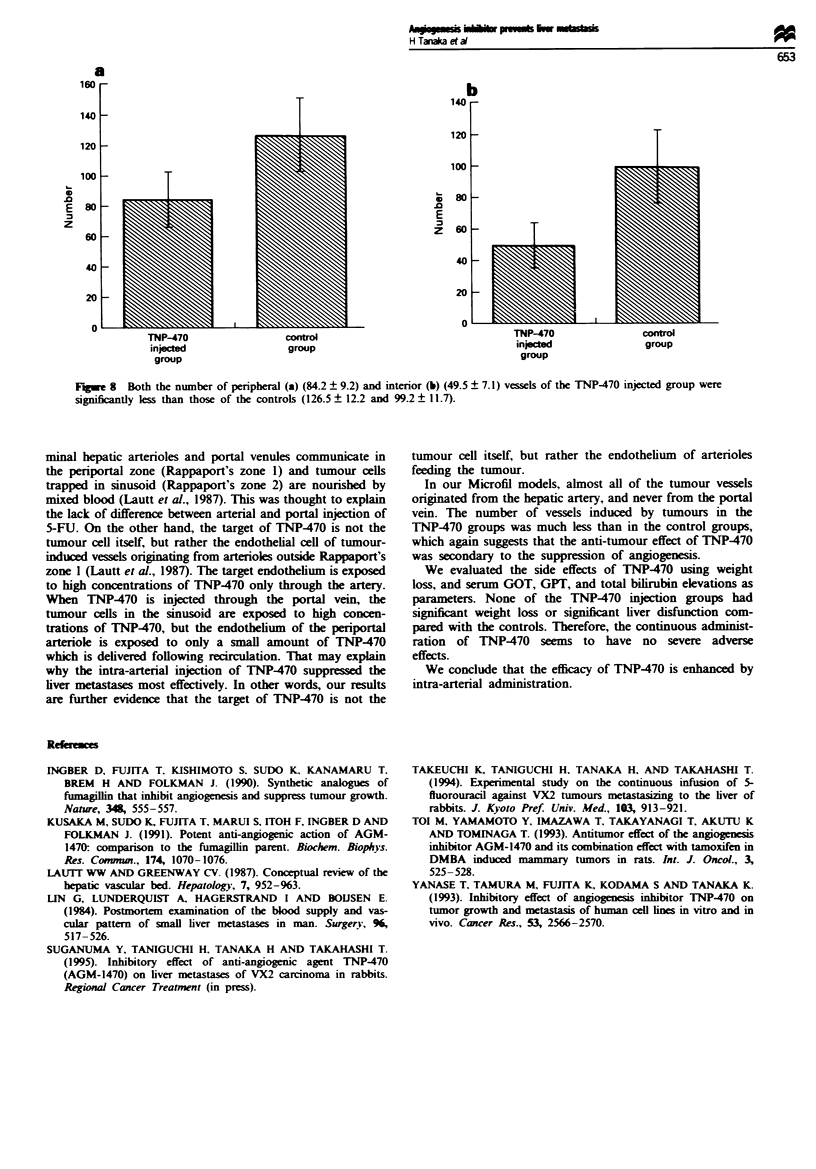# Intra-arterial administration of the angiogenesis inhibitor TNP-470 blocks liver metastasis in a rabbit model.

**DOI:** 10.1038/bjc.1995.389

**Published:** 1995-09

**Authors:** H. Tanaka, H. Taniguchi, T. Mugitani, Y. Koishi, M. Masuyama, T. Higashida, H. Koyama, Y. Suganuma, K. Miyata, K. Takeuchi

**Affiliations:** First Department of Surgery, Kyoto Prefectural University of Medicine, Japan.

## Abstract

**Images:**


British Journal of Cancer (1995) 72, 650-653
© 1995 Stockton Press All rights reserved 0007-0920/95 $12.00

### Intra-arterial administration of the angiogenesis inhibitor TNP-470 blocks liver metastasis in a rabbit model

H Tanaka, H Taniguchi, T Mugitani, Y Koishi, M Masuyama, T Higashida, H Koyama, Y Suganuma, K Miyata, K Takeuchi and T Takahashi

*First Department of Surgery, Kyoto Prefectural University of Medicine, Kyoto, Japan.*

**Summary** We evaluated the best route of administration of TNP-470, an angiogenesis inhibitor, by comparing the anti-tumour effects and toxicity following injection via the hepatic artery, the portal vein, or the jugular vein in a rabbit model of liver metastases. Following the injection of $1 \times 10^6$ VX2 carcinoma cells into the portal vein of rabbits, 50 mg of TNP-470 was injected continuously into the hepatic artery, portal vein, or jugular vein for 7 days. The number of tumours on the surface of the liver was counted 14 days following the start of the infusion, and the serum glutamic-oxaloacetic transaminase (GOT), glutamic-pyruvic transaminase (GPT) and total bilirubin concentrations were examined. In addition, a coloured silicon rubber was injected into the vessels of the liver to visualise the capillary networks around the tumours and assess the degree of suppression of angiogenesis by TNP-470. The mean number of tumours following intra-arterial injection ($17.5 \pm 2.9$) was significantly less than the control ($237.0 \pm 34.0$) ($P < 0.05$). The mean numbers of the tumours following intraportal ($89.1 \pm 16.0$) and intravenous ($140.6 \pm 31.2$) injection were both less than the controls ($215.3 \pm 45.5$, $284.8 \pm 55.4$ respectively), but the differences were not significant. We conclude that intra-arterial injection of TNP-470 is the most effective method for preventing liver metastases in this model.

**Keywords:** angiogenesis inhibitor; metastatic liver cancer; intra-arterial injection therapy

The prevention of haematogenous metastases, particularly to the liver, would profoundly improve the prognosis of most cancer patients. However, it is not yet known how to prevent or treat liver metastases. Chemotherapy usually is given for unresectable liver metastases, but several tumours are not sensitive to drugs currently available. Furthermore, most tumours develop resistance, and the toxicities of several agents are too great to permit prolonged treatment.

Because angiogenesis is essential for the proliferation of cancer cells, the inhibition of new blood vessel growth may be a useful way to prevent liver metastases (Ingber *et al.*, 1990). In addition, endothelial cells do not proliferate in adults except in a few parts of the genital organs (Kusaka *et al.*, 1991). Therefore, if one could inhibit the endothelial proliferation selectively, one could suppress tumour growth. A new synthetic analogue of fumagillin isolated from *Aspergillus fumigatus*, TNP-470, is known to inhibit angiogenesis by inhibiting DNA synthesis in endothelial cells selectively, and to have an anti-tumour effect (Ingber *et al.*, 1990; Kusaka *et al.*, 1991). We have already reported that intermittent intravenous injections of TNP-470 suppress the growth of VX2 tumour metastases in the livers of rabbits. In that study, the earlier TNP-470 was injected, the more effectively the liver metastases were suppressed (Suganuma *et al.*, in press). The purpose of the current study was to establish the best route of administration of TNP-470. We hypothesised that intra-arterial injection and intraportal injection would suppress liver metastases more effectively than would intravenous injection of this agent, based on our previous experiments with agents such as 5-fluorouracil (5-FU) (Takeuchi *et al.*, 1994). In addition, we visualised the small vessels generated around the liver tumours with a silicon rubber, to examine the angiogenesis inhibiting activity of TNP-470.

### Materials and methods

TNP-470 [*O*-(chloroacetyl-carbonyl)fumagilol] was obtained from Takeda Chemical Industries, Osaka, Japan. Thirty-four Japanese white rabbits weighing 2.0-3.0 kg were used for this study. VX2 carcinoma cells were maintained in the spleens of rabbits. VX2 tumours were broken into fine pieces, filtered through a stainless steel mesh, and suspended in Hanks' balanced salt solution (HBSS). The rabbits were anaesthetised by pentobarbital sodium ($30\ mg\ kg^{-1}$) which was given intravenously and subjected to laparotomy. VX2 carcinoma cells ($1.0 \times 10^6$) in 0.5 ml of HBSS were inoculated into the superior mesenteric vein to cause liver metastases. An ALZET osmotic pump, model 2ML1 (Alza Corporation, CA, USA), was used for the continuous infusion of TNP-470. The pump has a 2 ml capacity and can infuse an agent at a constant rate ($10\ \mu l\ h^{-1}$) for 7 days. A 50 mg aliquot of TNP-470 was dissolved in 2 ml of distilled water. Two ml of distilled water alone was used as a control.

The 34 rabbits were classified into three groups. The first received TNP-470 via the jugular vein (group TV: five rabbits), with a control group (group CV: five rabbits). A polyethylene catheter 1.22 mm in diameter (Becton Dickinson Labware, NJ, USA) was cannulated into the left jugular vein for both groups. Group TV received 50 mg of TNP-470 over 7 days (about $2.85\ mg\ kg^{-1}\ day^{-1}$), group CV received only 2 ml of distilled water into the vein over 7 days. The second group received TNP-470 via the portal vein (group TP: seven rabbits), with appropriate controls (Group CP: six rabbits). A 1.22 mm polyethylene catheter was inserted into the superior mesenteric vein and fitted with an ALZET pump. Group TP received 50 mg of TNP-470, and Group CP distilled water.

The third group received TNP-470 via the hepatic artery (group TA: eight rabbits), with controls, (group CA: seven rabbits). The polyethylene catheter was cannulated into the common hepatic artery from the left gastric artery, and the pump was attached. Group TA received 50 mg of TNP-470 into the hepatic artery, and group CA 2 ml of distilled water.

Fourteen days following the start of the infusion, blood was taken and analysed for serum GOT, GPT and total bilirubin concentrations. After being weighed, the rabbits were killed, and the number of tumours on the surface of the liver were counted macroscopically.

Just before being killed, each rabbit received 500 units $kg^{-1}$ heparin intravenously. Immediately following this, the liver was removed from the fresh cadaver, the portal vein and hepatic artery were exposed and cannulated with

Correspondence: H Tanaka, First Department of Surgery, Kyoto Prefectural University of Medicine, Kamigyo-ku, Kyoto 602, Japan
Received 30 November 1994; revised 9 March 1995; accepted 27 April 1995

polyethylene catheters. Microfil (Canton Bio-Medical Products, Boulder, CO, USA) was injected through the catheters until the vessels appeared well filled. Orange Microfil (MV-117) was used for the hepatic artery, and yellow (MV-122) was used for the portal vein (Lin *et al.*, 1984). Low infusion pressures were used to prevent extravasation of the Microfil. The specimens were refrigerated overnight to cure the Microfil. A part of each specimen was fixed in 10% formalin, sectioned at the maximal diameter of the tumours and stained by hematoxylin–eosin for histological examination. The number of vessels inside or around the tumours was then counted. The remainder of each specimen was immersed sequentially in 25%, 50%, 75%, 95%, and absolute ethanol for 1–2 days each, and finally immersed in methylsalicylate. The specimens became transparent and the vessels' structure could be examined visually with a stereoscope.

All data are expressed as means ± standard errors. The results were analysed by Student's *t*-test. A *P*-value $\leqslant 0.05$ was considered statistically significant.

**Figure 1** The number of tumours on the surface of each liver was counted macroscopically. The vertical lines show the 95% confidence limits. The number of tumours in the TA group was significantly less than the number of tumours in the CA group ($P<0.05$).

**Figure 2** Weight loss between the day of inoculation and 14 days later. There were no significant differences among the groups.

**Figure 3** The serum glutamic-oxaloacetic transaminase (GOT) concentration 14 days following the start of infusion. The mean GOT concentration in rabbits that received TNP-470 was not higher than that of the controls.

### Results

The mean number of tumours in group TA (17.5 ± 2.9) was significantly less than the number of tumours in the controls (237.0 ± 34.0) ($P<0.05$). The mean number of tumours in group TP (89.1 ± 16.0) and group TV (140.6 ± 31.2) were less than in the respective controls (215.3 ± 45.5, 284.8 ± 55.4), but were not significantly so. The mean number of tumours in Group TA was significantly less than in group TP or group TV ($P<0.05$) (Figure 1). None of the TNP-470 injection groups (TA: 0.16 ± 0.02 kg, TP: 0.12 ± 0.03 kg, TV: 0.19 ± 0.03 kg) exhibited significant weight loss compared with the controls (CA: 0.20 ± 0.03 kg, CP: 0.05 ± 0.01 kg, CV: 0.13 ± 0.04 kg (Figure 2). The serum GOT and GPT concentrations were not higher in the TNP-470 injected groups than in the controls (Figures 3 and 4). The total bilirubin was not elevated in any rabbit (Figure 5). Investigation under a stereoscope of the livers filled with Microfil revealed networks of vessels around tumours that originated mainly from the hepatic artery regardless of tumour size. The larger the tumour became, the more closely vessels grew around the tumour. However, the vessels around the tumours of the rabbits that received TNP-470 were less close than the vessels around similar sized tumours in the controls (Figure 6). Under histological examination, black material (Microfil) was observed in vessels, which were particularly dense around the tumours (Figure 7). Microfil was rarely seen in sinusoid, probably because it is too viscous to get into the sinusoid. No vessel was visible inside tumours less than 0.25 mm in diameter. Peripheral small necrotic areas and central necrosis were observed in some of the tumours over 1.75 mm in diameter in the TNP-470 injected groups and over 2.5 mm in diameter in the control groups. Most tumours over 4 mm in diameter had a large central necrotic zone.

Forty-one small metastases in the TP group and 38 in the CP group were examined for evaluation of capillary density. All of them were 0.25–4 mm in diameter because tumours of

**Figure 4** The serum glutamic–pyruvic transaminase (GPT) concentration 14 days following the start of infusion. There were no significant differences in the mean GPT concentration between the TNP-470 groups and the controls.

**Figure 5** The serum total bilirubin concentrations on the 14th day. No rabbit had a bilirubin over 1.0 mg $dl^{-1}$.

**Figure 6** Microfil filling the hepatic artery. The capillary networks around the tumours of the TA group (upper panels) were less dense than those of the controls (lower panels). Magnification × 6.6.

other sizes were unsuitable for counting the number of vessels inside the tumours. The number of cavities which were filled with Microfil and existed less than 0.25 mm from the tumour's edge and the number of cavities inside the tumours were counted individually. The number of peripheral (84.2 ± 9.2) and interior vessels (49.5 ± 7.1) of the TNP-470 injected group were significantly less than those of the controls (126.5 ± 12.2 and 99.2 ± 11.7) (Figure 8).

We suggested that the differences in the density of the capillary networks between the TNP-470 injected groups and the controls was caused by the angiogenesis inhibiting activity of TNP-470.

### Discussion

Kusaka *et al.* (1991) have reported that a broad range of concentrations of TNP-470, which is a synthetic analogue of fumagillin, a natural product of *Aspergillus fumigatus*, could selectively inhibit endothelial proliferation, with a subsequent anti-tumour effect. Several papers have previously described the anti-tumour effects of TNP-470, but most of them used a single systemic bolus injection of TNP-470 (Toi *et al.*, 1993; Yanase *et al.*, 1993).

As TNP-470 is cleared rapidly from the serum, we hypothesised that a continuous infusion of the drug would be more effective than intermittent administration. In spite of our expectations, continuous venous injection was no better than the intermittent injection reported previously. Today, chemotherapy for liver metastases is often administered through the hepatic artery. However, there is some evidence that in the first stage of metastasis, microemboli of tumour cells are nourished from the portal vein. It is controversial therefore whether the best route of administration of drugs to treat liver metastases is the portal vein or from the hepatic artery. In this study, the mean number of tumours in the intra-arterial injection group was significantly less than in the controls. The mean number of tumours in the intraportal and intravenous groups was less than in the controls, but the differences were not significant. Almost all the tumours were within 7 mm in diameter, with a few tumours in both the treated groups and control groups over 10 mm in diameter (maximum 15 mm). Although we expected the tumours to be smaller in the groups receiving TNP-470, this was not the case. We do not know why a few tumours in the treated groups could grow so large, but one hypothesis is that the agent is not delivered homogeneously into the entire liver.

**Figure 7** Microfil was filled into the vessels around and inside tumours, and is seen in black in this figure. Magnification × 40.

The current study indicates that arterial injection is the most effective route for treatment with TNP-470, which is different from our previous report using 5-FU for the same experimental liver metastases (Takeuchi *et al.*, 1994). In that study, continuous arterial and portal infusion of 5-FU were both more effective than systemic venous infusion, but there was no significant difference between arterial and portal administration of 5-FU. What caused this difference? Ter-

**Figure 8** Both the number of peripheral (**a**) (84.2 ± 9.2) and interior (**b**) (49.5 ± 7.1) vessels of the TNP-470 injected group were significantly less than those of the controls (126.5 ± 12.2 and 99.2 ± 11.7).

minal hepatic arterioles and portal venules communicate in the periportal zone (Rappaport's zone 1) and tumour cells trapped in sinusoid (Rappaport's zone 2) are nourished by mixed blood (Lautt *et al.*, 1987). This was thought to explain the lack of difference between arterial and portal injection of 5-FU. On the other hand, the target of TNP-470 is not the tumour cell itself, but rather the endothelial cell of tumour-induced vessels originating from arterioles outside Rappaport's zone 1 (Lautt *et al.*, 1987). The target endothelium is exposed to high concentrations of TNP-470 only through the artery. When TNP-470 is injected through the portal vein, the tumour cells in the sinusoid are exposed to high concentrations of TNP-470, but the endothelium of the periportal arteriole is exposed to only a small amount of TNP-470 which is delivered following recirculation. That may explain why the intra-arterial injection of TNP-470 suppressed the liver metastases most effectively. In other words, our results are further evidence that the target of TNP-470 is not the tumour cell itself, but rather the endothelium of arterioles feeding the tumour.

In our Microfil models, almost all of the tumour vessels originated from the hepatic artery, and never from the portal vein. The number of vessels induced by tumours in the TNP-470 groups was much less than in the control groups, which again suggests that the anti-tumour effect of TNP-470 was secondary to the suppression of angiogenesis.

We evaluated the side effects of TNP-470 using weight loss, and serum GOT, GPT, and total bilirubin elevations as parameters. None of the TNP-470 injection groups had significant weight loss or significant liver disfunction compared with the controls. Therefore, the continuous administration of TNP-470 seems to have no severe adverse effects.

We conclude that the efficacy of TNP-470 is enhanced by intra-arterial administration.

### References

INGBER D, FUJITA T, KISHIMOTO S, SUDO K, KANAMARU T, BREM H AND FOLKMAN J. (1990). Synthetic analogues of fumagillin that inhibit angiogenesis and suppress tumour growth. *Nature*, **348**, 555–557.

KUSAKA M, SUDO K, FUJITA T, MARUI S, ITOH F, INGBER D AND FOLKMAN J. (1991). Potent anti-angiogenic action of AGM-1470: comparison to the fumagillin parent. *Biochem. Biophys. Res. Commun.*, **174**, 1070–1076.

LAUTT WW AND GREENWAY CV. (1987). Conceptual review of the hepatic vascular bed. *Hepatology*, **7**, 952–963.

LIN G, LUNDERQUIST A, HAGERSTRAND I AND BOIJSEN E. (1984). Postmortem examination of the blood supply and vascular pattern of small liver metastases in man. *Surgery*, **96**, 517–526.

SUGANUMA Y, TANIGUCHI H, TANAKA H AND TAKAHASHI T. (1995). Inhibitory effect of anti-angiogenic agent TNP-470 (AGM-1470) on liver metastases of VX2 carcinoma in rabbits. *Regional Cancer Treatment* (in press).

TAKEUCHI K, TANIGUCHI H, TANAKA H, AND TAKAHASHI T. (1994). Experimental study on the continuous infusion of 5-fluorouracil against VX2 tumours metastasizing to the liver of rabbits. *J. Kyoto Pref. Univ. Med.*, **103**, 913–921.

TOI M, YAMAMOTO Y, IMAZAWA T, TAKAYANAGI T, AKUTU K AND TOMINAGA T. (1993). Antitumor effect of the angiogenesis inhibitor AGM-1470 and its combination effect with tamoxifen in DMBA induced mammary tumors in rats. *Int. J. Oncol.*, **3**, 525–528.

YANASE T, TAMURA M, FUJITA K, KODAMA S AND TANAKA K. (1993). Inhibitory effect of angiogenesis inhibitor TNP-470 on tumor growth and metastasis of human cell lines in vitro and in vivo. *Cancer Res.*, **53**, 2566–2570.